# Effect size and cost-effectiveness estimates of breast and cervical cancer screening reminders by population size through complete enumeration of Japanese local municipalities

**DOI:** 10.1186/1471-2458-14-43

**Published:** 2014-01-16

**Authors:** Shigekazu Komoto, Yuji Nishiwaki, Tomonori Okamura, Hideo Tanaka, Toru Takebayashi

**Affiliations:** 1 Department of Preventive Medicine and Public Health, School of Medicine, Keio University, 35 Shinanomachi, Shinjuku-ku, Tokyo 160-8582, Japan; 2 Department of Environmental and Occupational Health, Toho University, 5-21-16 Omori-nishi, Ota-ku, Tokyo 143-8540, Japan; 3 Division of Epidemiology and Prevention, Aichi Cancer Center Research Institute, 1-1 Kanokoden, Chikusa-ku, Nagoya, Aichi 464-8681, Japan

**Keywords:** Cancer screening, Reminder systems, Cost effectiveness, Breast cancer, Cervical cancer

## Abstract

**Background:**

Client reminders are known to increase cancer screening attendance rates. However, there are significant costs associated with them, and their effect by population size is unknown.

**Methods:**

In 2007 and 2008, the Japanese Government surveyed breast and cervical cancer screening in every municipality in Japan. From the results, we selected all 1,464 municipalities that carried out both screening types. We examined whether changes in screening attendance rates between 2007 and 2008 were associated with client reminders, number of public health nurses per 100,000 population, financial strength index, and 2007 attendance rates for different population sizes. We then calculated cost-effectiveness estimates of client reminders by population size and screening type.

**Results:**

Client reminders were associated with increased attendance rates in populations <100,000. For populations of 50,000–100,000, there was a 2.76% increase in breast cancer screening (95% CI: 0.41, 5.11), and a 2.25% increase in cervical cancer screening (95% CI: 0.89, 3.61). The incremental cost per additional attendance was higher in populations <50,000 than in populations of 50,000–100,000 (breast, $100 versus $54; cervical, $149 versus $67 respectively).

**Conclusions:**

Client reminders for breast and cervical cancer screening increased attendance rates in smaller municipalities in Japan.

## Background

Cancer is the leading cause of death in developed countries. Each year, breast and cervical cancers are responsible for the deaths of around 41,000 and 4,000 women in the United States and 12,000 and 1000 women in the United Kingdom, respectively [1,2]. Breast cancer is one of the most common cancers among Japanese women, and accounts for the death of around 12,000 women each year in Japan [3]. By contrast, the number of women in Japan who die of cervical cancer each year is only about 2,500 [3], but the increasing burden of this cancer in young women has a huge impact on society.

Many studies have reported that early detection and treatment of breast and cervical cancers could reduce their mortality [4-6]. Therefore, early detection screening programs are widely implemented in many countries. In Japan, screening guidelines recommend breast cancer screening (mammography examinations) for women aged over 40 years once every two years, and cervical cancer screening (cervical cytology testing) for women aged over 20 years once every two years. Japanese people are asked to make an effort to attend cancer screening. Despite this, breast and cervical cancer screening attendance rates are low. For example, only 24.3% of eligible women attended screening for either cancer in 2010 [7].

To reduce breast and cervical cancer mortality in Japan, increasing these low attendance rates has become an important issue. Based on the “Basic Law for the Promotion of Measures to Cope with Cancer,” the Ministry of Health, Labour and Welfare in Japan developed the “Plan to Cope with Cancer,” and one of the main goals of this plan was to raise cancer screening attendance rates to 50%. To help meet this target, municipalities in Japan are searching for effective measures to improve breast and cervical cancer screening attendance rates.

Earlier studies found that effective measures for improving screening attendance rates for these two types of cancers included client reminders and one-on-one education, with client reminders reported to have the largest effect [1,4,8-14]. However, the effectiveness of such measures may differ by population size, because that affects the residents’ sense of belonging and the community’s medical resources [15]. We therefore used survey data from all municipalities in Japan that conduct both breast and cervical cancer screening to assess the effect of client reminders on screening attendance rates, and to determine whether the effect varies by population size.

Each municipality is responsible for raising attendance rates in their respective areas. Because budgets are limited they have to use cost-effective methods to achieve this. It is therefore very important to estimate the effect of client reminders, together with an estimate of their cost per additional screening or to improve the percentage of the eligible population screened, by population size and separately for each type of cancer.

## Methods

### Cancer screening survey

The survey about cancer screening activity was carried out in 2007 and 2008 by the Japanese Ministry of Health, Labour and Welfare (MHLW) [16,17]. It asked the administrative office of each municipality to report whether or not they carried out cancer screening, how they selected their target residents, and whether or not they sent reminders. In both years, all municipalities (1,822 in 2007 and 1,818 in 2008) responded to the survey, a response rate of 100%. For inclusion in our study, municipalities had to have carried out both breast and cervical cancer screening in 2007 and 2008, and not have merged with another municipality during the period.

Based on whether or not client reminders were issued, we divided the municipalities into four groups. Group 1 did not issue client reminders in 2007 or 2008. Group 2 issued client reminders in 2007 but not in 2008. Group 3 did not issue client reminders in 2007 but did in 2008. Group 4 issued client reminders in both 2007 and 2008.

The subjects of this study were not individuals but every municipality in Japan, so no ethical consent was needed.

### Screening attendance rate estimation

We used the equation below to estimate the 2007 and 2008 screening attendance rates in each municipality.

$$\mathrm{Cancer}\;\mathrm{screening}\;\mathrm{attendance}\;\mathrm{rate}\;\left(\%\right)=\left[\left(\mathrm{number}\;\mathrm{of}\;\mathrm{cancer}\;\mathrm{screening}\;\mathrm{tests}\;\mathrm{conducted}\;\mathrm{in}\;\mathrm{municipality}\right)/\left(\mathrm{number}\;\mathrm{of}\;\mathrm{subjects}\;\mathrm{in}\;\mathrm{municipality}\;\mathrm{eligible}\;\mathrm{for}\;\mathrm{cancer}\;\mathrm{screening}\right)\right]\times 100$$

The numerator (number of cancer screening tests conducted in municipality) was extracted from statistical survey data collected every year by the MHLW [18,19]. The denominator (number of subjects in municipality eligible for cancer screening) for breast cancer was the total female population aged 40 or older, and for cervical cancer, it was the total female population aged 20 or older in each municipality [20]. Those who were offered cancer screening by their workplace, so would not have their test conducted in the municipality, and would therefore not appear in the numerator, were also excluded from the denominator.

### Covariates

We obtained the number of public health nurses in each municipality from statistical survey data [18,19] collected every year by the MHLW, and calculated the number of public health nurses per 100,000 people. We used the Ministry of Internal Affairs and Communications’ statistical surveys to obtain each municipality’s population size and financial capability index. The financial capability index is used to indicate the financial strength of local public entities, and is calculated from the figures provided by each municipality as the most recent three-year average of the figures derived from dividing basic financial earnings by basic financial needs [21,22].

### Statistical analysis

We calculated the differences in attendance rates between 2007 and 2008 for breast and cervical cancer screening, and then the average difference for each group to identify whether sending reminders made any difference to attendance. The average difference was compared among the groups by using a multiple regression analysis. The adjusted differences and corresponding 95% confidence intervals were calculated for each group, with group 1 as a reference. We also looked for correlations between attendance rate and various covariates, including the attendance rate in 2007 (continuous), the number of public health nurses per 100,000 population, and the financial strength index. We divided the latter two into four equal categories for analysis. We repeated the regression analysis for different population sizes. According to the Local Government Act in Japan, municipalities with populations less than 50,000 are defined as “towns”, and larger municipalities as “cities”. The median population size among cities is 104,321 and so we further divided cities into two almost equal groups using a population point of 100,000, giving us three groups for population size: “less than 50,000”, “between 50,000 and 100,000”, and “more than 100,000”.

### Cost-effectiveness calculations

The primary outcomes of the cost-effective calculations are values for “increased number of cancer screening attendees” and “increased attendance rate for cancer screening” in municipalities where client reminders were carried out in 2008 but not in 2007. We estimated the cost per single additional attendee and of increasing the attendance rate by 1% for each population size category and for the two types of cancer.

Municipalities generally inform their residents about cancer screening using media such as public information magazines and their website. However, no data was collected about these more general methods. We therefore could not compare the cost-effectiveness of individual client reminders with different alternative interventions, or estimate the effect of the more general methods.

The measured outcomes were “changes in numbers of cancer screening attendees” and “changes in rate of cancer screening attendance” in the year of the intervention, which is a more direct and immediate measure of success than mortality rates. Earlier studies have already proved that cancer screening can reduce the mortality rate for breast cancer and cervical cancer [4-6], so the purpose of this study is to identify suitable ways in which to increase attendance, rather than to reduce mortality.

The cost of carrying out client reminders is the total cost of posting individual reminders to all eligible residents. The cost of posting one individual reminder is 150 yen ($1.5 US, at exchange rates published on 5 September 2013) (this includes paper, printing, envelope and personnel expenses at 70 yen, and a postage fee of 80 yen). It is considered that every municipality could issue client reminders using the existing postage service, without incurring any other costs.

To estimate the cost of increasing the attendance rate by 1% for each population size category, we estimated the cost of the 25th and 75th percentile population size in each category. Because we investigated the short-term change in attendance rates in the same year as an intervention, we did not need to apply any discounting.

For the sensitivity analysis, we estimated the cost of increasing attendance by one person and increasing the attendance rate by 1% for each population size category and for the two types of cancer.

## Results

The distributions of population size, numbers of public health nurses and financial capability index groups by client reminder group are shown in Table 1. There were 644 (44.0%) municipalities that did not issue client reminders in 2007. Of those, 168 issued client reminders in 2008.

**Table 1:** Population, number of public health nurses and financial capability index by client reminder group

			**Client reminder group**								**Total N = 1,464**	
		**Median**	**Group 1 (2007 no–2008 no) N = 476**		**Group 2 (2007 yes–2008 no) N = 200**		**Group 3 (2007 no–2008 yes) N = 168**		**Group 4 (2007 yes–2008 yes) N = 620**			
			**n**	**%**	**n**	**%**	**n**	**%**	**n**	**%**	**n**	**%**
Population	Fewer than 50,000	14,926	306	64.3%	128	64.0%	125	74.4%	422	68.1%	981	67.0%
	Between 50,000 and 100,000	67,181	88	18.5%	34	17.0%	17	10.1%	97	15.6%	236	16.1%
	More than 100,000	189,735	82	17.2%	38	19.0%	26	15.5%	101	16.3%	247	16.9%
Number of public health nurses	Group 1 (≤16.0/100,000)	11.5	159	33.4%	60	30.0%	36	21.4%	143	23.1%	398	27.2%
	Group 2 (16.1–26.1/100,000)	20.7	124	26.1%	42	21.0%	38	22.6%	162	26.1%	366	25.0%
	Group 3 (26.2–43.3/100,000)	32.4	83	17.4%	48	24.0%	46	27.4%	176	28.4%	353	24.1%
	Group 4 (≥43.4/100,000)	64.8	110	23.1%	50	25.0%	48	28.6%	139	22.4%	347	23.7%
Financial capability index	Group 1 (≤0.28)	0.21	108	22.7%	44	22.0%	48	28.6%	118	19.0%	318	21.7%
	Group 2 (0.29–0.48)	0.38	98	20.6%	54	27.0%	45	26.8%	171	27.6%	368	25.1%
	Group 3 (0.49–0.73)	0.60	127	26.7%	49	24.5%	36	21.4%	161	26.0%	373	25.5%
	Group 4 (≥0.74)	0.93	143	30.0%	53	26.5%	39	23.2%	170	27.4%	405	27.7%

Significant correlations were identified between client reminders and population size (p = 0.015), number of public health nurses per population (p < 0.001) and financial capability index (p = 0.030).

Table 2 shows breast and cervical cancer screening attendance rates in 2007 and 2008 for each reminder group, as well as the differences in attendance rates between 2007 and 2008. Table 3 shows the results of the multivariate analysis using the difference in attendance rates as the dependent variable. When group 1 was used as the reference, groups 3 and 4 had a larger increase in attendance rates even after adjusting for covariates, with the largest increase in group 3 (reminders introduced in 2008) for both breast and cervical cancer screening. When we ran the analysis by population size, we observed a statistically significant increase in the attendance rates of groups 3 and 4 (compared with group 1) for breast cancer, and for group3 for cervical cancer in populations of less than 50,000 and in the rates of group 3 for both in populations between 50,000 and 100,000. The effect size was largest in populations between 50,000 and 100,000 (2.76% for breast cancer [95% CI: 0.41, 5.11], 2.25% for cervical cancer [95% CI: 0.89, 3.61]).

**Table 2:** Breast and cervical cancer screening attendance rates in 2007 and 2008

		**Client reminder group**								**Total N = 1,464**	
		**Group 1 (2007 no–2008 no) N = 476**		**Group 2 (2007 yes–2008 no) N = 200**		**Group 3 (2007 no–2008 yes) N = 168**		**Group 4 (2007 yes–2008 yes) N = 620**			
**Breast cancer screening**											
Attendance rate in 2007*	Median (IQR)	8.14	(5.43–12.04)	9.28	(6.57–12.64)	10.68	(6.81–18.11)	11.89	(8.09–16.88)	10.10	(6.52–14.88)
Attendance rate in 2008*	Median (IQR)	7.66	(5.20–11.44)	8.64	(5.71–11.52)	10.72	(6.40–17.80)	11.38	(7.7–16.52)	9.71	(6.04–14.56)
Difference	Mean, Standard Deviation	-0.17	4.06	-1.31	7.40	-0.26	5.27	-0.44	6.30	-0.45	5.74
**Cervical cancer screening**											
Attendance rate in 2007*	Median (IQR)	9.49	(6.54–12.93)	11.18	(8.42–15.82)	13.36	(8.36–19.90)	14.29	(9.84–20.54)	11.85	(8.12–17.41)
Attendance rate in 2008*	Median (IQR)	9.23	(6.65–12.62)	10.23	(7.88–15.39)	13.18	(8.20–19.39)	14.52	(9.79–20.11)	11.62	(7.98–17.11)
Difference	Mean, Standard Deviation	-0.21	2.88	-0.69	3.27	0.22	4.24	-0.26	4.04	-0.25	3.63

*p < 0.001 (Kruskal-Wallis test).

**Table 3:** Factors associated with changes in breast and cervical cancer screening attendance rates by population size

			**Total**			**Population size**								
						**Fewer than 50 000**			**Between 50 000 and 100 000**			**More than 100 000**		
			**Adjusted difference**	**95% CI**		**Adjusted difference**	**95% CI**		**Adjusted difference**	**95% CI**		**Adjusted difference**	**95% CI**	
**Breast cancer screening**														
	Reminders	Group 1 (2007 no–2008 no)	-	-		-	-		-	-		-	-	
		Group 2 (2007 yes–2008 no)	-0.60	(-0.46, 0.26)		-0.42	(-1.62, 0.77)		-0.43	(-2.20, 1.34)		-0.92	(-2.11, 0.28)	
		Group 3 (2007 no–2008 yes)	1.24	(0.32, 2.16)	**	1.50	(0.28, 2.72)	*	2.76	(0.41, 5.11)	*	-0.25	(-1.65, 1.14)	
		Group 4 (2007 yes–2008 yes)	1.13	(0.49, 1.77)	**	1.31	(0.43, 2.18)	**	0.72	(-0.64, 2.07)		0.84	(-0.10, 1.78)	
	2007 Attendance rates		-0.35	(-0.39, -0.31)	**	-0.38	(-0.43, -0.33)	**	-0.30	(-0.40, -0.20)	**	-0.20	(-0.29, -0.12)	**
	Number of public health nurses	Group 1 (≤16.0/100 000)	-			-			-			-		
		Group 2 (16.1–26.1/100 000)	0.70	(-0.07, 1.48)		0.68	(-0.76, 2.13)		0.20	(-1.11, 1.51)		-0.92	(-1.17, 0.99)	
		Group 3 (26.2–43.3/100 000)	1.01	(0.14, 1.89)	*	0.94	(-0.53, 2.41)		0.62	(-1.21, 2.46)		-0.88	(-2.80, 2.63)	
		Group 4 (≥43.4/100 000)	2.02	(0.99, 3.05)	**	1.94	(0.38, 3.51)	*	1.98	(-6.83, 10.79)		-		
	Financial strength index	Group 1 (≤0.28)	-			-			-			-		
		Group 2 (0.29–0.48)	0.62	(-0.27, 1.51)		0.59	(-0.42, 1.60)		0.11	(-8.58, 8.81)		-		
		Group 3 (0.49–0.73)	1.21	(0.23, 2.19)	*	1.10	(-0.08, 2.29)		1.69	(-7.07, 10.44)		-1.10	(-2.76, 0.56)	
		Group 4 (≥0.74)	1.62	(0.60, 2.63)	**	2.20	(0.92, 3.49)	**	1.45	(-7.32, 10.22)		-0.97	(-2.60, 0.65)	
**Cervical cancer screening**														
	Reminders	Group 1 (2007 no–2008 no)	-			-			-			-		
		Group 2 (2007 yes–2008 no)	-0.15	(-0.73,0.43)		-0.03	(-0.84, 0.78)		-0.25	(-1.30, 0.80)		-0.41	(-1.34, 0.51)	
		Group 3 (2007 no–2008 yes)	0.95	(0.32, 1.57)	**	1.01	(0.18, 1.84)	*	2.25	(0.89, 3.61)	**	-0.37	(-1.44, 0.70)	
		Group 4 (2007 yes–2008 yes)	0.49	(0.06, 0.93)	*	0.54	(-0.06, 1.14)		0.64	(-0.13, 1.42)		0.20	(-0.54, 0.94)	
	2007 Attendance rates		-0.11	(-0.13, -0.09)	**	-0.11	(-0.14, -0.08)	**	-0.10	(-0.15, -0.05)	**	-0.13	(-0.18, -0.08)	**
	Number of public health nurses	Group 1 (≤16.0/100 000)	-			-			-			-		
		Group 2 (16.1–26.1/100 000)	0.10	(-0.41, 0.62)		0.26	(-0.73, 1.24)		0.04	(-0.73, 0.81)		-0.76	(-1.60, 0.07)	
		Group 3 (26.2–43.3/100 000)	0.84	(0.26, 1.42)	**	1.05	(0.06, 2.05)	*	0.00	(-1.07, 1.08)		-0.26	(-2.36, 1.84)	
		Group 4 (≥43.4/100 000)	1.40	(0.71, 2.09)	**	1.58	(0.52, 2.64)	**	0.30	(-4.86, 5.47)		-		
	Financial strength index	Group 1 (≤0.28)	-			-			-			-		
		Group 2 (0.29–0.48)	0.69	(0.09, 1.29)	*	0.61	(-0.08, 1.30)		-0.01	(-5.13, 5.10)		-		
		Group 3 (0.49–0.73)	1.38	(0.71, 2.04)	**	1.38	(0.57, 2.18)	**	0.06	(-5.09, 5.20)		-0.29	(-1.57, 0.99)	
		Group 4 (≥0.74)	2.19	(1.50, 2.88)	**	2.67	(1.80, 3.54)	**	0.72	(-4.43, 5.87)		-0.10	(-1.35, 1.15)	

*p < 0.05, **p < 0.01 (two-sided).

The degree of increase in attendance rate was associated with the previous level of attendance, i.e. the higher the attendance rate in 2007, the smaller the increase in attendance rate. As the number of public health nurses per 100,000 population increased, the attendance rate changed more markedly. In the same way, as the financial capability index categories increased, the attendance rate changed more markedly. For both cancers, both these associations were strongest in municipalities with populations smaller than 50,000.

The cost-effectiveness estimates are shown in Table 4. The incremental cost per additional screening in municipalities with populations fewer than 50,000 was $100 for breast cancer screening and $149 for cervical cancer screening. For populations between 50,000 and 100,000, the costs were estimated at $54 for breast cancer screening and $67 for cervical cancer screening. Looking at the data another way, the inter-quartile range for the cost of increasing the attendance rate by 1% was from $2,041 to $8,408 for breast cancer screening, and $3,030 and $12,487 for cervical cancer screening in municipalities with populations fewer than 50,000. In municipalities with populations between 50,000 and 100,000, it was between $9,229 and $12,867 for breast cancer screening, and $11,321 and $15,783 for cervical cancer screening. This suggests that breast cancer screening reminders were more cost-effective than cervical cancer screening reminders.

**Table 4:** Cost-effectiveness of improving screening rates by sending out patient reminders by population size

			**Population (n)**	**Estimated number of eligible subjects **^**a**^**(n)**	**Cost of posting a reminder to one person(US dollars**^**b**^**/person)**	**Cost of posting reminders to all subjects (thousand US dollars**^**b**^**)**	**Attendance rate improvement attributed to patient reminders**^**c **^**(%)**	**Increase in the number of screening tests attributed to patient reminders (n)**	**Incremental cost per additional screening (US dollars**^**b**^**)**	**Cost of increasing attendance rate by 1% (US dollars**^**b**^**)**
Breast cancer screening	Population fewer than 50 000	25%	6,917	2,041	1.5	3.1	1.5	31	100	2,041
		Median	14,926	4,403	1.5	6.6	1.5	66	100	4,403
		75%	28,501	8,408	1.5	12.6	1.5	126	100	8,408
Population between 50 000 and 100 000		25%	57,566	16,982	1.5	25.5	2.76	469	54	9,229
		Median	67,181	19,818	1.5	29.7	2.76	547	54	10,771
		75%	80,255	23,675	1.5	35.5	2.76	653	54	12,867
Cervical cancer screening	Population fewer than 50 000	25%	6,917	2,041	1.5	3.1	1.01	21	149	3,030
		Median	14,926	4,403	1.5	6.6	1.01	44	149	6,539
		75%	28,501	8,408	1.5	12.6	1.01	85	149	12,487
Population between 50 000 and 100 000		25%	57,566	16,982	1.5	25.5	2.25	382	67	11,321
		Median	67,181	19,818	1.5	29.7	2.25	446	67	13,212
		75%	80,255	23,675	1.5	35.5	2.25	533	67	15,783

^a^Breast cancer screening is offered to women older than 40 years (29.5% of the population), and cervical cancer screening to women older than 20 years (42.3% of the population).

^b^1 US dollar =100 yen.

^c^Attendance rate improvement attributed to patient reminders was calculated in Table 3.

## Discussion

From our findings, it seems likely that client reminders can increase breast and cervical cancer screening attendance rates, especially in municipalities with populations fewer than 100,000. Client reminders were more cost-effective in improving breast cancer screening attendance rates than cervical cancer screening attendance rates. The marginal cost of increasing attendance ranged from $54 to $149, depending on population size and cancer type.

Individual reminders are recommended by the US Task Force on Community Preventive Services to improve breast and cervical cancer screening attendance rates [1]. Our results support this recommendation, particularly for municipalities with populations fewer than 100,000. A recent systematic review revealed that printed reminders could increase breast cancer screening attendance rates by 3.6% (IQI = 1.8, 14.0) [8]. The attendance rate improvement (1.24%) observed across all municipalities in this study falls below this. However, the 2.76% increase observed in municipalities with populations between 50,000 and 100,000 is closer to that figure.

For cervical cancer screening attendance rates, in our study, an improvement of 0.95% was observed across all municipalities, and an improvement of 2.25% was observed in municipalities with populations between 50,000 and 100,000. These figures fall well short of that estimated for cervical cancer in the systematic review (10.2%, IQI = 6.3, 17.9) [8]. There are two possible reasons for the smaller effect of client reminders observed in our study. First, it is possible that the risk of cervical cancer is not common knowledge among those in their 20s, because the target population for cervical cancer screening in Japan did not include women younger than 30 years old until 2004. This could result in a smaller increase in the cervical cancer screening attendance rate. Second, the client reminders were mainly sent via post in Japan, whereas some of the reminders examined in the systematic review were issued by phone instead, which might have greater impact.

Client reminder effects were seen in small but not large municipalities. This could be because strong social networks promote medical check-ups in small municipalities [15]. The sense of ‘belonging’ is higher among residents of small municipalities, and they are therefore more likely to have a stronger response to encouragement from their municipality. In addition, in large municipalities, there are many medical institutions where residents can freely receive consultations. Residents may therefore use cancer screening services provided by these medical institutions, instead of the services provided by the municipality. Such cancer screenings were not counted in our survey, because they were not provided by municipalities. Our study could therefore be underestimating both the actual numbers of screenings and the efficacy of reminders in these large municipalities.

A previous study showed that sending out individual or customized reminder letters improved cervical cancer screening attendance rates by 9.4%, whereas no significant improvement was seen when routine reminder letters were sent [9]. Customized reminders may have been included among those issued by municipalities in our survey, but this information was not collected. We may be able to increase the effectiveness of reminders by customizing them, but it is extremely difficult to create individually-customized reminders, and there are few cancer screening experts in the municipalities of Japan to do so.

In municipalities in Japan, public health nurses conduct cancer screening. They are also in charge of various services such as infant health check-ups and providing measures against infectious diseases like tuberculosis. Our results show that the more public health nurses per population, the stronger the effect of the reminders in increasing cancer screening attendance rates. This could simply be the effect of more appointments and clinics being available for cancer screening, or it could be related to increased familiarity with, and therefore acceptance of, the role of public health nurses, leading to increased likelihood of eligible women taking up the opportunity for screening tests. Further investigation would be necessary to establish the precise links between numbers of nurses per population and cancer screening increases.

The financial burden on municipalities of providing cancer screening services is large. Our results suggest that wealthier municipalities may devote more of their budget to cancer screening, and that this is then associated with higher attendance rates.

An intervention study of 6,133 women, carried out in North West London and the West Midlands in the United Kingdom, reported that the incremental cost per additional breast cancer screening of sending reminders by post was £26 (approximately $41.8, at current exchange rates) [12]. In our study, in municipalities with populations between 50,000 and 100,000, the cost per additional breast cancer screening was $54. However, in the UK study, the attendance rate in the non-intervention group was 55.3%, and in the intervention group was 64.4%, which is five times higher than Japanese attendance rates, and thus cannot easily be compared with our study.

This study was strengthened by the strong reliability of the information sources and the 100% response rate of all municipalities conducting cancer screening in Japan. Furthermore, the information about client reminders and covariate data were obtained separately from attendance rates, strengthening the data. However, this study has several limitations. We were unable to investigate the effects of individual methods such as post versus telephone reminders, because the details of the client reminder methods used by each municipality were unclear. We were also unable to investigate the effect of personal characteristics on screening attendance, because we did not have any information on individual residents such as past consultation history, educational background, and income. For instance, the effect of reminders in the form of letters is reported to be stronger in groups who have previously had a mammogram or cervical cancer cytology test than in those who have never been screened [10,11]. In this study, we could not investigate whether this was the case because of the lack of information on past consultation history. If this was true, we believe that it could be possible to increase cost-effectiveness by only sending out reminders to those who have a previous history of consultations [4,10,13]. However, such an approach, although perhaps more cost-effective, would have considerable ethical issues, and could not be advocated without an alternative method being used to reach out to those who had not previously attended. By collecting more information about individuals and reminder methods, we could investigate more effective ways to increase participation in cancer screening.

Japan's screening attendance rate is much lower than those of Western countries, so our results are thought to be more applicable to regions with low attendance rates, and to countries that aim to implement cancer screening in the future.

This study provides valuable information to staff in charge of cancer screening tests in municipalities, to help them decide whether to issue client reminders. On the basis of our findings, we recommend that they should do so, especially in municipalities with population size up to 100,000, and for breast cancer screening.

## Conclusions

In conclusion, we saw an improvement in the 2008 breast and cervical cancer screening attendance rates in municipalities that issued client reminders. The improvements were most obvious in municipalities with populations fewer than 100,000. Improving breast cancer screening attendance rates was more cost-effective than improving cervical cancer screening rates.

## Competing interests

The authors declare that they have no competing interests.

## Authors’ contributions

KS conducted the statistical analysis and drafted the manuscript. NY and OT contributed to the design of the study, data analysis and interpretation of the analysis. TH and TT were involved in interpretation of the analysis and critical revision of manuscript. All authors have read, edited and approved the final manuscript.

## Pre-publication history

The pre-publication history for this paper can be accessed here:

http://www.biomedcentral.com/1471-2458/14/43/prepub
